# Perceived Food Insecurity, Dietary Quality, and Unfavorable Food Intake among Children and Adolescents from Economically Disadvantaged Households

**DOI:** 10.3390/nu13103411

**Published:** 2021-09-27

**Authors:** Chiu-Wen Yeh, Yuan-Ting C. Lo, Yi-Chieh Chen, Wei-Chih Chen, Yi-Chen Huang

**Affiliations:** 1 Department of Nutrition, China Medical University, No. 100, Sec. 1, Jingmao Rd., Taichung 406040, Taiwan; u108076201@cmu.edu.tw (C.-W.Y.); s110355@shsh.tw (Y.-C.C.); 2 Department of Public Health, National Defense Medical Center, No. 161, Sec. 6, Minquan E. Rd., Neihu Dist., Taipei 11490, Taiwan; yuantinglo@gmail.com; 3 Boyo Social Welfare Foundation, No. 131, An 4th St., Puli Township, Nantou County 54547, Taiwan; chen157@ecp.boyo.org.tw

**Keywords:** food security, dietary diversity score, dietary quality, children, adolescents

## Abstract

Few studies have investigated food security, dietary quality, or unfavorable food intake through self-reports among children and adolescents in Asia. This study assessed the association of perceived food insecurity with dietary quality and unfavorable food intake among 1111 children and 538 adolescents from economically disadvantaged families in Taiwan. Food security status was collected by a validated questionnaire. Dietary quality was examined using a dietary diversity score (DDS). Unfavorable food intake was defined as fried food, bread/cake/pastries, sugar-sweetened beverages, and biscuits/chips. Food-insecure participants had lower DDS, whole grains and protein-rich food intake than food-secure participants. Furthermore, food-insecure children had a higher frequency of unfavorable food consumption. The level of children’s food insecurity was inversely associated with DDS (β: −0.047, 95% CI: −0.085 to −0.009) but positively with bread/pastry (β: 0.103, 95% CI: 0.022–0.184) and sugar-sweetened beverages (β: 0.117, 95% CI: 0.018–0.215) intake. Adolescents who reported food insecurity and not enough money for household expenses had an odds ratio of 2.85 (95% CI: 1.15–7.10) for poor DDS relative to their food-secure and financially able counterparts. We recommended that health policy needs to include diversifying food and nutrition education for vulnerable children and adolescents to improve dietary quality.

## 1. Introduction

The elimination of hunger is one of the Sustainable Development Goals for 2030 proposed by the United Nations [1]. The principle of food security is to ensure that every individual has an affordable access to safe and quality diet [2]. However, nutritional policies to end hunger have mainly focused on increasing food availability, and limited data are available on diet quality [1]. A healthy diet should be both sufficient and diverse for physiological and psychological development in childhood and adolescence.

The prevalence of global food insecurity in 2020 increased from 26.6% to 30.4% compared to 2019, with the rate increasing from 22.7% to 25.8% for the Asian region, during the coronavirus (COVID-19) pandemic [3]. The impact of the pandemic has led to difficulties in food transportation, increased food prices, and loss of income, which has increased the risk of food insecurity among children and adolescents from economically disadvantaged households [4,5]. In 2009, 22.0% of Taiwanese adolescents from economically disadvantaged families faced two or more types of food insecurity problems [6], but the current status is unknown. Therefore, exploring food security and its association with dietary quality and unfavorable food intake during the COVID-19 pandemic could provide information that enables health departments to prioritize children and adolescents’ health requirements and integrate our health and welfare system.

The association between food insecurity and dietary quality has been inconsistent in children and adolescents in Western countries [7] and is unknown in Asian populations. Food-insecure children tend to have unhealthy dietary patterns of consuming more high-energy-dense but low-nutrient-dense food, and partake in less physical activity [7,8], which increases the risk of obesity [9]. This may be because a healthier diet is more expensive, forcing them to buy low-cost but high-energy and low-nutrient-dense foods in the circumstances [10]. Furthermore, economically disadvantaged children and adolescents may encounter more barriers to developing healthy eating behaviors, such as parents or caregivers with low or limited health literacy. A study revealed that food-insecure children, but not adolescents, eat more home-cooked food, legumes, eggs, and ultra-processed food [11].

The transition period from childhood to adolescence also marks the transition of independence of food intake, from being dependent on caregivers to developing nutritional autonomy [12]. Dietary quality decreases from childhood to adolescence, probably because the determinants of food choice vary with age, such as a decrease in parental control, influence by peers, and autonomy of food choice, which increase with age [12,13]. This could be due to the difference between childhood and adolescence.

Thus, personal eating behavior and the environment may be more critical factors affecting dietary quality in adolescents than household food insecurity [11,12]. Therefore, it is important to explore the influence of household economic status on the association between food security and the dietary quality of children and adolescents.

Moreover, the experiences of food insecurity in children or adolescents are typically obtained by interviewing their parents or caregivers, which may not be accurate as they may not know what the children or adolescents eat outside the home. Indeed, the agreement between children and parents regarding children’s food insecurity is only 21.7%, and parents tend to underreport it [14]. In Taiwan, the average duration of school and after-school activities for children and adolescents is 9.5 h (7:30 am to 5:00 pm), which is different from other countries. Therefore, the perceived physiology and emotional responses of facing food insecurity obtained directly from children and adolescents are more reliable indicators than parent response.

In this study, we assessed the prevalence of food insecurity among children and adolescents from economically disadvantaged families in Taiwan during the COVID-19 pandemic and investigated the association of food insecurity with dietary quality and unfavorable food intake.

## 2. Materials and Methods

### 2.1. Participants

This was a cross-sectional study based on the Food Security Survey from September to October 2020. The participants were recruited from the nonprofit organization Boyo Social Welfare Foundation, which provides free after-school programs for elementary school students (age 7–12 years) and junior high school students (age 13–15 years) from low-income families. Low-income families were defined as households with limited resources and income less than the poverty line of the local area [15]. All children and adolescents were evaluated by the Boyo Social Welfare Foundation through household environment visits, and their average monthly distributable income was NT$5500–NT$6500 (approximately US$180–US$212) per person after deducting all household expenses [16]. A total of 1668 participants from 16 centers completed the questionnaire. We excluded 19 participants with missing data related to food security, and included 1649 (1111 children [aged 7–12 years and going to elementary school] and 538 adolescents [aged 13–15 years and going to junior high school]) for the analysis. We collected their demographic, dietary intake, and food security data using a self-reported questionnaire. Our staff assisted and explained the questionnaire if the participants did not understand the question. This study’s protocol was approved by the Central Regional Research Ethics Committee of China Medical University (CRREC-109-099). Written informed consent was obtained from the parents of all participants.

### 2.2. Measurements

#### 2.2.1. Dietary Intake

Dietary quality was evaluated by using the dietary diversity score (DDS) [17,18]. DDS comprises six food groups based on Taiwan Dietary Guidelines: whole grain, vegetables, fruits, dairy, soy/fish/egg/meat, and oil and nuts. The DDS considers not only dietary diversity but also the minimal amounts they eat. Intake of each food group was counted as achieving one-half serving and was assigned 1 point, a total of 6 points. A higher DDS indicates better dietary quality and diversity. DDS presents a straightforward way to measure nutrient adequacy, nutrient intake, and nutritional status [19]. We used a half-serving food image to assist the participant in recalling and answering the question. Good or poor dietary quality was defined as a score of ≥4 or <4; this cut point was considered a good indicator for simple food quality and had a positive relationship with health [17].

A simple food frequency questionnaire was used to evaluate four types of unhealthy foods: fried food, bread/cake/pastries, sugar-sweetened beverages (SSB), and biscuits/potato chips. One of the following six responses could be selected: (1) Did not eat or less than once a week; (2) Once a week; (3) 2–4 times per week; (4) 5–6 times per week; (5) Once a day; and (6) ≥2 times a day. We counted the frequency of these intakes per week. We also evaluate the household food supply through the following question: “Has your family bought the following foods in the past month?” for 45 food items, which were selected by referring to Nutrition and Health Survey in Taiwan [20].

#### 2.2.2. Food Security

Questionnaires were used to evaluate the food security status in children and ad-olescents for considering their cognition development. Children’s experiences of food insecurity were examined by using a self-report 5-item Child Food Security Assessment. This assessment comprised three domains (cognitive, emotional, and physical awareness of food security) and was developed by Fram et al.; it has been validated for use in young children (age: 7 years) [8,21]. Each question asked children “how frequently they had experienced situations of food insecurity over the past month”. For the response options of “never,” “one or two times,” and “many times,” the score was 0, 1, and 2, respectively [8]. The range of score was 0–10, and a higher score indicated a more frequently experience of food insecurity. Cronbach’ α is 0.704.

Adolescents’ experiences of food insecurity were examined using a 9-item self-report questionnaire developed by Connell et al. [22]. The questions are about “their home food security status during the last month”, and the responses are rated as “a lot,” “sometimes,” and “never.”, the score was 1, 1, and 0, respectively [22]. The score range was 0–9, with a higher score indicating more severe food insecurity. Cronbach’ α is 0.874. Food insecurity is indicated by a score of ≥2 for both question-naires and details of questionnaires can be found elsewhere [8,14,21,22]. 

### 2.3. Statistical Analysis

All data analyses were performed using SAS (version 9.4, SAS Institute, Cary, NC, USA). Data were presented as mean ± standard deviation and number (%) for continuous and category variables, respectively. Student’s t test and chi-square test were used to evaluate the difference between baseline characteristics and food security by children and adolescents. Multiple linear regression was used to evaluate the β coefficients (95% confidence interval) for the association of food security with dietary quality and unfavorable food intake. The β coefficients were expressed as the changes in DDS or frequency of unfavorable food intake (dependent variables) for every increase in the food insecurity score (independent variable). Covariates were selected into the adjusted model when a significant difference (*p* < 0.005) was noted between baseline characteristics and food security or DDS. Age and body mass index (BMI) were adjusted based on the previous studies [23,24]. A series of analyses was conducted by adjusting for demographic variables (base model), household variables (model 2), and household financial status (model 3) to clarify the association between dependent and independent variables. Model 1 was adjusted for age, sex, body mass index (underweight, normal weight, overweight, and obese), and region (East, North, Central, South, and Outlying Island). Model 2 included additional adjustments for number of children in household, number of household members, father’s nationality, father’s education level (elementary school, junior high school, senior high school, and college or above). Model 3 included additional adjustments for whether own the house (yes, no), household expense whether enough (enough, just enough, and not enough), and weekly allowance (NT$0, <NT$50, NT$51–100, and ≥NT$100). No collinearity was found among the independent variables by using the variance inflation factor (all value <2). The joint effect of food security, household expense whether enough and DDS scores was calculated.

## 3. Results

### 3.1. Baseline Characteristics by Food Security

Table 1 presents the distribution of baseline characteristics based on food security in children and adolescents. The prevalence of having experienced food insecurity was 57.9% and 40.5% in children and adolescents, respectively. Food-insecure children and adolescents tended to have a less educated father (children: 6.7% vs. 4.3%; adolescents: 6.0% vs. 4.7%), and not enough household expenses (children: 19.4% vs. 12.4%; adolescents: 21.1% vs. 5.6%). Moreover, food-insecure children tended to live in the north of Taiwan (27.4% vs. 22.9%), had higher weekly allowance (21.8% vs. 15.8%), had more children in the household (3.15 ± 1.58 vs. 2.95 ± 1.40), and had more household members (6.08 ± 2.51 vs. 5.78 ± 2.13) (all *p* < 0.05). In contrast, adolescents who had food insecure experiences tended to be male (54.1% vs. 45.0%), had lower weekly allowances (44.5% vs. 50.9%), and had Taiwanese fathers (95.4% vs. 98.8%) (all *p* < 0.05).

### 3.2. Food Intake and Unfavorable Food Intake by Food Security

**Figure 1:**
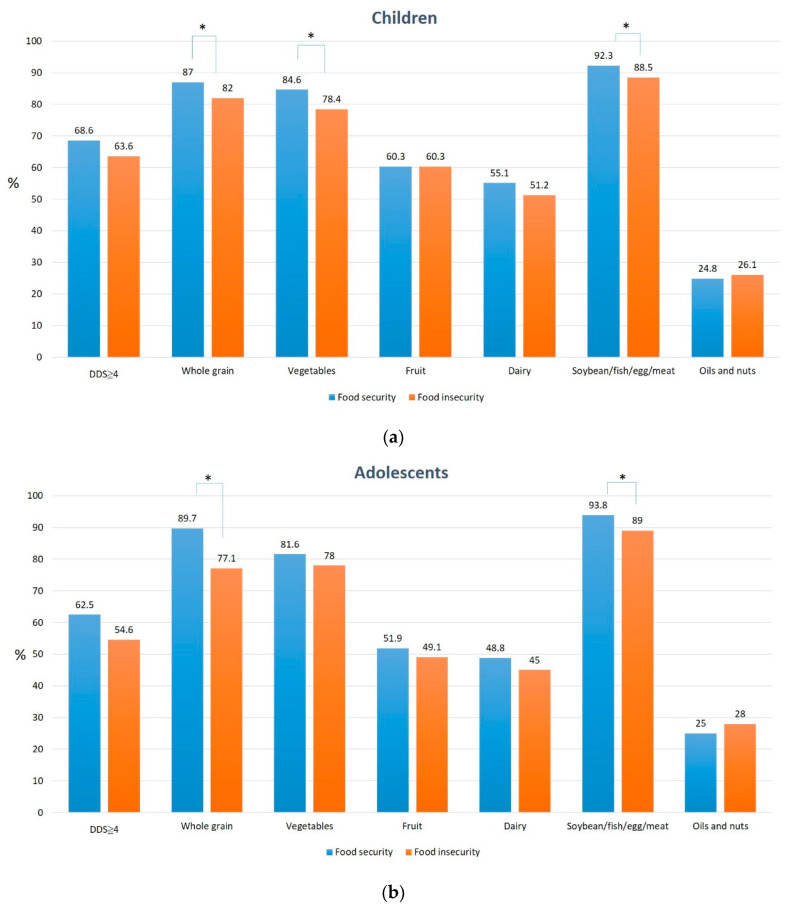
Dietary diversity score and its components and food security among children (**a**) and adolescents (**b**). * *p* < 0.05.

**Figure 2:**
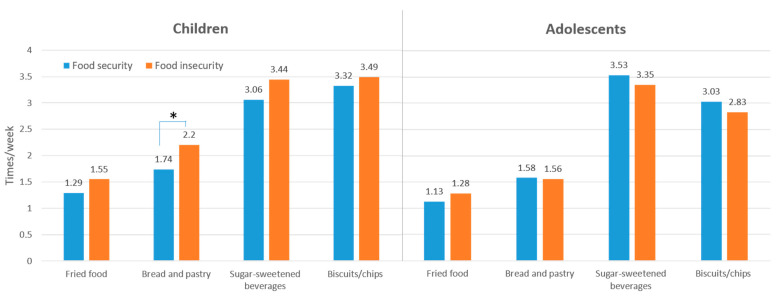
Frequency of unfavorable food intake for children and adolescents. * *p* < 0.05.

Food-insecure children and adolescents had significantly lower DDS than food-secure children and adolescents, respectively (children: 3.86 ± 1.41 vs. 4.04 ± 1.36, *p* = 0.037; adolescents: 3.66 ± 1.42, 3.91 ± 1.33, *p* = 0.041). Figure 1 illustrates food intake by food security in children (Figure 1a) and adolescents (Figure 1b). Food-insecure children and adolescents had a lower percentage of achieving one-half serving of whole grain (children: 82.0% vs. 87.0%, *p* = 0.024; adolescents: 77.1% vs. 89.7%, *p* < 0.0001) and soybean/fish/egg/meat intake (children: 88.5% vs. 92.3%, *p* = 0.036; adolescents: 89.0% vs. 93.8%, *p* = 0.048). Moreover, food-insecure-children tended to have a significantly lower percentage of achieving one-half serving of vegetables (78.4% vs. 84.6%, *p* = 0.009) but had a higher intake of bread and pastry (2.20 vs. 1.74 times/week, *p* = 0.011), compared with food-secure children. However, no difference was observed between unfavorable food intake and food security status among adolescents (Figure 2).

### 3.3. Association between Food Security and Dietary Quality and Unfavorable Food Intake

Table 2 presents the β coefficients (95% CIs) of the association between food security score and DDS and the frequency of unfavorable food intake. In the crude model, the level of food insecurity was associated with lower DDS among children (β = −0.044, 95% CI: −0.081 to −0.008) and adolescents (β = −0.052, 95% CI: −0.099 to −0.004). In contrast, the level of food insecurity was associated with increased frequency of fried food (β = 0.085, 95% CI: 0.016–0.154), bread/pastry (β = 0.115, 95% CI: 0.037–0.193), and SSB (β = 0.154, 95% CI: 0.057–0.251) intake among children; however, no such significant associations were observed in adolescents. After adjusting for potential confounders and financial variables (whether own the household, whether household expenses were enough, and allowance), the level of children’s food insecurity remained associated with lower DDS (β = −0.047, 95% CI: −0.085 to −0.009), but positively associated with fried food (β = 0.071, 95% CI: −0.0007 to 0.142), bread/pastry (β = 0.103, 95% CI: 0.022 to 0.184) and SSB intake (β = 0.117, 95% CI: 0.018 to 0.215) (model 3). For adolescents, the level of food insecurity was significantly inversely associated with DDS (β = −0.050, 95% CI: −0.099 to −0.001) (model 2). However, further adjustment for financial variables showed the significant association among adolescents no longer existed (β = −0.047, 95% CI: −0.098 to 0.005) (model 3). Furthermore, no association was observed with unfavorable food intake in adolescents.

### 3.4. Joint Effect of Food Security and Dietary Quality by Household Financial Status

We further explored the influence of household financial status on the association between food security and dietary quality. The joint effect of food security and DDS based on whether there is enough money for household expenses is presented in Table 3. In all, 21.1% of adolescents were food insecure and perceived that they did not have enough money for household expenses, and had an odds ratio of 2.85 (95% CI: 1.15–7.10) for poor DDS (<4) compared with food-secure adolescents with enough money for the household expenses (model 2). After further adjustment for house ownership and allowance, the odds ratio (95% CI) was 2.33 (95% CI: 0.92–5.92) (model 3), but it was not significant. The children had non-significant patterns.

## 4. Discussion

We observed that the prevalence of having experienced food insecurity was 57.9% and 40.5% among Taiwanese children and adolescents from economically disadvantaged families. Furthermore, children’s level of food insecurity was inversely associated with dietary quality and positively with unfavorable food intake during the COVID-19 pandemic. In addition, food insecure adolescents with the most critical financial problem household had two-fold risk of poor dietary quality.

Dietary diversity is an indicator of household food security and is part of a healthy diet [19]. Our results revealed that dietary diversity is associated with perceived food security among children. A diverse diet ensures sufficient nutrient intake and is linked to growth and health. In this study, food-insecure children had lower intake of whole grains, vegetables, and protein-rich foods. Grain is the primary energy source, vegetables provide vitamins and phytochemicals, and protein-rich food ensures their physical development.

Story et al. [13] and other researchers [12] have proposed many factors affecting children and adolescents’ food choices based on social cognitive theory and ecological models. In childhood, food choice and intake are mainly controlled by physiology (hunger), family environment (home food availability), and social environment (socioeconomic position). Studies from developed countries have revealed that children from low-income families tend to consume high-energy-dense and low-nutrient-dense foods [11,26]. Fram et al. reported that children’s food insecurity was associated with lower vegetable intake but higher energy, fat, and sugar intake by using the same child self-report questionnaire in the United States [8]. These foods are categorized as ultraprocessed foods, such as SSBs and potato chips [27]. They provide food palatability and satiety at a lower cost than whole foods [28]. Our results are consistent with these findings. Furthermore, food-insecure children tend to consume more processed and ultraprocessed food than food-secure children; it may be related to their household purchase, which is low in milk, fresh vegetables, fruit, fish, and seafood but high in milk powder, processed fruit, and processed meat (Supplementary Table S1). In Taiwan, milk, vegetables, fruits, and meat are more expensive than other food groups. A Swedish study demonstrated that children’s healthy diet is linked to higher diet costs partly due to the price differences between healthy and less-healthy foods [29]. Furthermore, high-calorie foods may be cheaper because low-calorie-dense foods such as vegetables or fruits are required to be purchased at a higher cost to achieve satiety [10]. Therefore, households with a limited budget tend to choose low-cost and long-shelf-life foods, thereby might influence children’s dietary variety and its quality contents.

Our data indicated that food insecurity was not associated with dietary diversity and unfavorable food intake among adolescents after adjustment for financial status. These findings may be explained by the effect of the family food environment, which is lower in adolescents than in children, whereas social environment—food preferences, appeal of food, convenience, school food environment, peer influences, and food advertising—plays a role in adolescent’s diet. Adolescents spend a longer time outside home and have higher autonomy to prepare or purchase foods [12]. Food intake, mainly ultraprocessed foods, from outside of home increases with age [30,31]. The cost and convenience of the external school food environment are key factors influencing adolescents’ food choices [32]. Our data also revealed that food-insecure adolescents reported a higher percentage of hunger than food-secure adolescents (5.1% vs. 0.3%) due to a shortage of allowance to purchase food. Allowance is a proxy to present the autonomy of food choice when eating out [33]. In addition, not enough household financial status was associated with poor dietary quality among adolescents, but not in children (data not shown). Therefore, the association between food insecurity and dietary diversity may confounded by household financial status and allowance among adolescents. Adolescents’ autonomy and independence need to be balanced to maintain health by consuming diverse foods with a limited food budget and further investigation study is needed.

In our study, the most vulnerable group was adolescents with perceived food insecurity and insufficient ability to pay for household expenses (with the highest risk of DDS < 4). These adolescents had less educated parents, unemployed parents, more family members, less pocket money, and poorer food accessibility (data not shown). Our government has been providing economic and social welfare for members of a low-income or middle-to-low-income, however, it might have overlooked the food benefit in terms of individual needs. Several strategies for health and nutritional policies might be considered among economically disadvantaged adolescents. Thus, nutrition education is urgently required to promote inexpensive yet healthy food selection among adolescents. We strongly recommend working with non-governmental organizations (NGOs) to provide adequate assistance.

This study has the following strengths. We measured food security by self-reporting, which can reduce the bias of misclassification of food insecurity. Furthermore, the large sample size enabled us to investigate the association between food security, dietary diversity, and unfavorable food intake in children and adolescents from economically disadvantaged families. However, this study also has some limitations. First, we cannot rule out the possibility that siblings were included in the analysis and may overestimate the association. Second, we did not collect information on who received the food aid or participated in the food programs. It may have led to an underestimation of the prevalence of food insecurity.

## 5. Conclusions

This study observed that food-insecure is inversely associated with dietary quality among economically disadvantaged children and adolescents in Taiwan. Further, the level of food insecurity was positively associated with unfavorable food intake for children. The most vulnerable group (food insecurity and household financial difficulty) had a higher risk of poor dietary quality among adolescents. This study confirmed that health and nutritional policy not only provides food subsidies but also needs to consider diversifying food and nutrition education, mainly in adolescents for improving food choice. Furthermore, cooperation with NGOs is required to meet the possible individual needs.

## Figures and Tables

**Table 1:** Baseline characteristics of participants by food security among children and adolescents. (*n* = 1649).

	Children (7–12 Years Old, *n* = 1111)			Adolescents (13–15 Years Old, *n* = 538)		
	Food Security	Food Insecurity	*p* Value ^1^	Food Security	Food Insecurity	*p* Value ^1^
Total	468 (42.1)	643 (57.9)		320 (59.5)	218 (40.5)	
***Participant’s characteristics***						
Age (yrs)	9.76 ± 1.56	9.85 ± 1.50	0.359	13.51 ± 0.84	13.53 ± 0.87	0.807
Gender (% boys)	220 (47.0)	304 (47.3)	0.929	144 (45.0)	118 (54.1)	0.038
Body mass index (BMI) ^2^			0.583			0.850
Underweight	35 (7.6)	57 (9.0)		21 (6.7)	14 (6.5)	
Normal weight	261 (56.4)	340 (53.6)		205 (64.9)	134 (61.8)	
Overweight	73 (15.8)	93 (14.7)		35 (11.1)	25 (11.5)	
Obesity	94 (20.3)	144 (22.7)		55 (17.4)	44 (20.3)	
Region			0.029			0.154
East	18 (3.9)	24 (3.7)		9 (2.8)	10 (4.6)	
North	107 (22.9)	176 (27.4)		62 (19.4)	58 (26.6)	
Central	213 (45.5)	290 (45.1)		123 (38.4)	77 (35.3)	
South	93 (20.0)	129 (20.1)		74 (23.1)	48 (22.0)	
Island	37 (7.9)	24 (3.7)		52 (16.3)	25 (11.5)	
Allowance (NTD/week)			0.020			0.021
None	216 (46.2)	244 (38.0)		67 (20.9)	36 (16.5)	
<50	89 (19.0)	134 (20.8)		21 (6.6)	29 (13.3)	
51–100	89 (19.0)	125 (19.4)		69 (21.6)	56 (25.7)	
≥101	74 (15.8)	140 (21.8)		163 (50.9)	97 (44.5)	
***Household’s characteristics***						
Father’s nationality (% of Taiwan)	460 (98.3)	634 (98.6)	0.678	316 (98.8)	208 (95.4)	0.017
Father’s education level			0.031			0.007
Elementary school	20 (4.3)	43 (6.7)		15 (4.7)	13 (6.0)	
Junior high school	81 (17.3)	146 (22.7)		69 (21.7)	64 (29.4)	
Senior high school	194 (41.5)	252 (39.2)		157 (49.1)	72 (33.0)	
College or above	56 (12.0)	76 (11.8)		20 (6.3)	15 (6.9)	
Unknown	117 (25.0)	126 (19.6)		59 (18.4)	54 (24.8)	
Mother’s nationality (% of Taiwan)	386 (82.5)	548 (85.2)	0.217	224 (70.0)	164 (75.2)	0.184
Mother’s education level			0.104			0.727
Elementary school	24 (5.1)	53 (8.2)		29 (9.1)	20 (9.2)	
Junior high school	81 (17.3)	133 (20.7)		63 (19.7)	50 (22.9)	
Senior high school	176 (37.6)	224 (34.8)		123 (38.4)	84 (38.5)	
College or above	79 (16.9)	108 (16.8)		30 (9.4)	14 (6.4)	
Unknown	108 (23.1)	125 (19.4)		75 (23.4)	50 (22.9)	
Number of children	2.95 ± 1.40	3.15 ± 1.58	0.024	2.73 ± 1.48	2.88 ± 1.44	0.249
Number of household member	5.78 ± 2.13	6.08 ± 2.51	0.036	5.39 ± 2.03	5.31 ± 2.13	0.643
Live in own house	309 (66.0)	437 (68.0)	0.497	242 (75.6)	147 (67.4)	0.037
Whether had enough money for household expense			0.0005			<0.0001
Enough	89 (19.0)	82 (12.8)		57 (17.8)	12 (5.5)	
Just enough	321 (68.6)	436 (67.8)		245 (76.6)	160 (73.4)	
Not enough	58 (12.4)	125 (19.4)		18 (5.6)	46 (21.1)	

Data were presented as *n* (%) or mean ± SD. ^1^ Comparsion of the demographic characteristics by food security status. *p* was test by the chi-square test for categorical variables and Student t test for continuous variables. ^2^ Cut-off points of BMI for age and gender-specific based on recommended by the Health Promotion Administration [25].

**Table 2:** β coefficients (95% confidence interval) of association between food security status and dietary quality score, unfavorable food intake by multiple linear regression.

	Crude Model	Model 1	Model 2	Model 3
Children (*n* = 1111)				
Dietary diversity score	−0.044 (−0.081, −0.008) *	−0.046 (−0.084, −0.009) *	−0.046 (−0.084, −0.009) *	−0.047 (−0.085, −0.009) *
Frequency of unfavorable food intake				
Fried food	0.085 (0.016, 0.154) *	0.082 (0.012, 0.152) *	0.079 (0.008, 0.149) *	0.071 (−0.0007, 0.142)
Bread and pastry	0.115 (0.037, 0.193) **	0.108 (0.029, 0.187) **	0.104 (0.024, 0.184) *	0.103 (0.022, 0.184) *
Sugar sweetened beverages	0.154 (0.057, 0.251) **	0.146 (0.049, 0.243) **	0.133 (0.035, 0.231) **	0.117 (0.018, 0.215) *
Biscuits and snack	0.085 (−0.013, 0.183)	0.084 (−0.015, 0.184)	0.072 (−0.029, 0.172)	0.065 (−0.036, 0.166)
Adolescents (*n* = 538)				
Dietary diversity score	−0.052 (−0.099, −0.004) *	−0.055 (−0.103, −0.007) *	−0.050 (−0.099, −0.001) *	−0.047 (−0.098, 0.005)
Frequency of unfavorable food intake				
Fried food	0.020 (−0.045, 0.084)	0.029 (−0.037, 0.094)	0.031 (−0.037, 0.099)	0.031 (−0.040, 0.102)
Bread and pastry	−0.003 (−0.080, 0.086)	0.012 (−0.072, 0.097)	0.006 (−0.080, 0.093)	0.022 (−0.069, 0.113)
Sugar sweetened beverages	−0.066 (−0.189, 0.056)	−0.056 (−0.181, 0.069)	−0.071 (−0.199, 0.057)	−0.059 (−0.192, 0.075)
Biscuits and snack	−0.049 (−0.162, 0.065)	−0.012 (−0.126, 0.102)	−0.019 (−0.136, 0.099)	0.028 (−0.094, 0.151)

β coefficient (95% CI) analyzed using generalized linear models. Model 1: adjusted for gender, age, body mass index, and region. Model 2: adjusted for covariates in model 1 plus number of siblings, number of household members, father’s nationality, father’s education level. Model 3: adjusted for covariates in model 2 plus whether own the household, household expense whether enough, and allowance. * *p* < 0.05, ** *p* < 0.01.

**Table 3:** Odds ratio (95% CI) of poor dietary quality (DDS < 4) by joint effect of food security and household financial status among children and adolescents.

	Food Security			Food Insecurity		
	Whether Had Enough Money for Household Expense					
	Enough	Just Enough	Not Enough	Enough	Just Enough	Not Enough
Children						
Crude model	1	1.07 (0.64, 1.78)	1.03 (0.51, 2.12)	0.95 (0.49, 1.83)	1.39 (0.85, 2.26)	1.34 (0.75, 2.39)
Model 1	1	1.18 (0.70, 1.98)	1.24 (0.59, 2.58)	1.04 (0.54, 2.03)	1.50 (0.91, 2.49)	1.54 (0.85, 2.49)
Model 2	1	1.09 (0.64, 1.85)	1.16 (0.55, 2.44)	1.03 (0.53, 2.21)	1.42 (0.86, 2.37)	1.43 (0.78, 2.62)
Model 3	1	1.06 (0.62, 1.81)	1.10 (0.52, 2.34)	1.03 (0.52, 2.01)	1.40 (0.84, 2.34)	1.38 (0.75, 2.55)
Adolescents						
Crude model	1	2.68 (1.35, 5.31) **	1.88 (0.58, 6.00)	1.88 (0.48, 7.30)	3.15 (1.55, 6.39) **	3.44 (1.45, 8.13) **
Model 1	1	2.48 (1.24, 4.97) *	1.89 (0.58, 6.16)	2.00 (0.50, 7.98)	3.10 (1.51, 6.35) **	3.39 (1.41, 8.14) **
Model 2	1	2.38 (1.18, 4.81) *	1.99 (0.60, 6.61)	2.03 (0.50, 8.16)	3.03 (1.46, 6.31) **	2.85 (1.15, 7.10) *
Model 3	1	2.25 (1.11, 4.57) *	1.77 (0.53, 5.89)	1.93 (0.48, 7.78)	2.88 (1.38, 6.02) **	2.33 (0.92, 5.92)

Odds ratio (95% CI) using logistic regression model. Model 1: adjusted for gender, age, body mass index, and region. Model 2: adjusted for covariates in model 1 plus number of siblings, number of household members, father’s nationality, father’s education level. Model 3: adjusted for covariates in model 2 plus whether own the household, and allowance. * *p* < 0.05, ** *p* < 0.01.

## Data Availability

The data presented in this study are available on request from the corresponding author. The data are not publicly available due to privacy issue.

## Supplementary Materials

The following are available online at https://www.mdpi.com/article/10.3390/nu13103411/s1, Table S1: title: Percentage of household food items ever purchased in the past month.
