# Supplementary material for: Perceived Food Insecurity, Dietary Quality, and Unfavorable Food Intake among Children and Adolescents from Economically Disadvantaged Households
Source: Nutrients. 2021 Sep 27;13(10):3411. doi: 10.3390/nu13103411 (PMC8540399; doi:10.3390/nu13103411)
Supplement: Supplementary file 1 [file nutrients-13-03411-s001.zip › nutrients-1362704-supplementary.pdf]

Supplementary Table S1. Percentage of household food items ever purchased in the past month

	Children (N=1111)			Adolescent (N=538)		
	Food security (N=468)	Food insecurity (N=643)	*p* value	Food security (N=320)	Food insecurity (N=218)	*p* value
Dairy products						
Milk	356 (76.1)	447 (69.5)	0.016	265 (82.8)	164 (75.2)	0.032
Milk powder	93 (19.9)	136 (21.2)	0.603	68 (21.3)	47 (21.6)	0.932
Flavored milk	156 (33.3)	230 (35.8)	0.400	114 (35.6)	76 (34.9)	0.856
Vegetable and fruit						
Fresh vegetable	385 (82.3)	473 (73.6)	0.0006	251 (78.4)	180 (82.6)	0.239
Fresh fruit	381 (81.4)	490 (76.2)	0.037	263 (82.2)	166 (76.2)	0.087
Dried fruit, preserves, jam	85 (18.2)	142 (22.1)	0.109	46 (14.4)	39 (17.9)	0.273
Canned fruit	44 (9.4)	86 (13.4)	0.042	16 (5.0)	17 (7.8)	0.184
Not 100% fruit juice	90 (19.2)	130 (20.2)	0.684	65 (20.3)	48 (22.0)	0.634
Grains						
Rice	415 (88.7)	565 (87.9)	0.681	285 (89.1)	196 (89.9)	0.754
Pasta, rice noodles	309 (66.0)	380 (59.1)	0.019	189 (59.1)	128 (58.7)	0.936
Protein-rich food						
Pork	302 (64.5)	388 (60.4)	0.155	243 (75.9)	153 (70.2)	0.137
Chicken	315 (67.3)	409 (63.6)	0.201	257 (80.3)	166 (76.2)	0.247
Fish	341 (72.9)	417 (64.9)	0.005	231 (72.2)	164 (75.2)	0.433

Seafood	226 (48.3)	251 (39.0)	0.002	171 (53.4)	94 (43.1)	0.019
Egg	357 (76.3)	471 (73.3)	0.252	265 (82.8)	175 (80.3)	0.454
Processed meat products	201 (43.0)	318 (49.5)	0.032	169 (52.8)	86 (39.5)	0.002
Others						
Instant noodle	297 (63.5)	408 (64.5)	0.998	215 (67.2)	126 (57.8)	0.027
SSB	323 (69.0)	440 (68.4)	0.835	246 (76.9)	163 (74.8)	0.575
Breads, sweets and snacks	265 (56.6)	331 (51.5)	0.089	183 (57.2)	112 (51.4)	0.184
Fried food	227 (48.5)	334 (51.9)	0.258	145 (45.3)	97 (44.5)	0.852
Donuts/Muffins/crepes	97 (20.8)	180 (28.0)	0.006	55 (17.2)	29 (13.3)	0.223
